# The development of the PlexiQoL: A patient‐reported outcome measure for adults with neurofibromatosis type 1‐associated plexiform neurofibromas

**DOI:** 10.1002/mgg3.1530

**Published:** 2020-10-21

**Authors:** Alice Heaney, Jeanette Wilburn, Matthew Rouse, Shannon Langmead, Jaishri O. Blakeley, Susan Huson, Stephen P. McKenna

**Affiliations:** ^1^ Galen Research Manchester UK; ^2^ Johns Hopkins Comprehensive Neurofibromatosis Clinic Baltimore MD USA; ^3^ Manchester Centre for Genomic Medicine Saint Mary’s Hospital Manchester UK; ^4^ School of Health Sciences The University of Manchester Manchester UK

**Keywords:** neurofibromatosis type 1, patient‐reported outcome measure, plexiform neurofibromas, PlexiQoL, quality of life

## Abstract

**Background:**

To develop and validate a patient‐reported outcome (PRO) measure of quality of life (QoL), specific to patients with Neurofibromatosis Type 1 (NF1)‐associated plexiform neurofibromas (pNFs), suitable for use in clinical efficacy trials. The study was conducted in parallel in the UK and US.

**Methods:**

Qualitative interviews were conducted with patients to generate questionnaire content. Face and content validity of the draft scale was assessed by cognitive debriefing interviews (CDIs). A postal validation survey was conducted to identify the final version of the questionnaire (the PlexiQoL), establish its unidimensionality, and assess its psychometric properties.

**Results:**

Thematic analysis was performed on 42 interview transcripts. Thirty‐one CDIs revealed that patients found the draft scale to be comprehensible, relevant, and easy to complete. The postal validation survey was completed by 273 patients. Rasch analysis identified an 18‐item unidimensional scale that showed excellent internal consistency, reproducibility, and sensitivity to differences in patient‐perceived pNF severity, general health, and the use of pain medication.

**Conclusions:**

The PlexiQoL is the first disease‐specific PRO assessing the ability of adults with NF‐1 associated pNFs to meet their basic human needs. Clinical trials are planned to assess the sensitivity to change of the PlexiQoL in people undergoing treatment for pNFs.

## Graphical abstract

The aim of this study was to develop and validate a patient‐reported outcome (PRO) measure of quality of life (QoL), specific to patients with Neurofibromatosis Type 1 (NF1)‐associated plexiform neurofibromas (pNFs), suitable for use in clinical efficacy trials. Rasch analysis identified an 18‐item unidimensional scale that showed excellent internal consistency, reproducibility and sensitivity to differences in patient‐perceived pNF severity, general health and the use of pain medication.

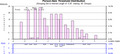

## 1 INTRODUCTION

Neurofibromatosis type 1 (NF1) is an autosomal dominant genetic disorder which affects roughly 1 in 3,000 people globally (Children’s Tumor Foundation, 2018). Approximately 25,000 people in the UK (The Neuro Foundation, 2018) and over 100,000 people in the US are currently affected (Neurofibromatosis Network, 2018). Estimates of the proportion of people with NF1 who have pNFs vary from one third (Darrigo et al., 2007) to a half (Evans et al., 2003; Kim et al., 2009; Mautner et al., 2008).

The impact of pNFs can be highly variable. In some individuals the effect can be minimal, while others struggle with disfigurement, pain, neurological dysfunction, psychological stress, or functional disability (Vranceanu et al., 2013). These effects may be present for much of a person's life due to the early onset of the condition (Tucker et al., 2009). Furthermore, there is a 10% lifetime risk of pNFs undergoing malignant transformation (Evans et al., 2003). Thus, pNFs can have a profound impact on a person's quality of life (QoL).

The current treatment paradigm for these tumors is either symptom management or surgical reduction. However, surgery often has limited success due to the high risk of nerve damage, the inability to completely resect and, consequently, a high rate of recurrence (Dombi et al., 2016). There are programs dedicated to the acceleration of treatment developments for pNFs, with preliminary clinical trials yielding promising results in terms of improving radiographic tumor response associated with pNFs (Blakeley & Plotkin, 2016). However, radiographic improvement of clinical characteristics does not directly translate to patient benefit. To determine the true value of a treatment, it is necessary to ask the “experts” – the people living with the condition.

Patient‐reported outcomes (PROs) are a valuable tool for this purpose, and they are increasingly being used as endpoints in clinical trials (Gnanasakthy et al., 2012). Two main types of PRO are used in clinical research; health‐related quality of life (HRQL) and needs‐based quality of life (QoL). The former is concerned primarily with factors that are directly influenced by health services, such as symptoms and functional limitations. Rather than measuring symptoms and functioning directly, QoL assesses the impact of these (and additional relevant influences) on the overall life of the patient. The needs‐based model states that QoL is dependent on a person's ability to meet his or her basic human needs. QoL is poor when few needs are fulfilled (Hunt & McKenna, 1992). As HRQL and QoL measures assess different types of outcome, they can be used in combination, rather than being considered alternatives.

Valid PROs must meet strict criteria if they are to be used in clinical research. These criteria include having a clear theoretical basis, content derived from patients, acceptability to respondents, and strong psychometric properties (Dirven et al., 2018; U.S. Department of Health & Human Services FDA Center for Drug Evaluation & Research, U.S. Department of Health & Human Services FDA Center for Biologics Evaluation & Research, U.S. Department of Health & Human Services FDA Center for Devices & Radiological Health, 2006). Disease‐specific measures derived from the needs‐based model of QoL satisfy these fundamental requirements (Wilburn et al., 2015, 2017).

Clinical studies of NF1 have used the SF‐36 (Ware & Sherbourne, 1992) and the Skindex (Chrsen et al., 1996). As these are generic HRQL measures, many of their items may not be relevant to NF1 patients with pNFs or are likely to miss important, specific issues. Furthermore, both measures were developed prior to the application of new statistical techniques in health outcomes research, such as Rasch Measurement Theory (RMT; Rasch, 1960), which improves the precision of measurement (Prieto et al., 2003; Wright, 1996). Valid PRO measurement requires a coherent conceptual model of the outcome being measured, content derived directly from relevant patients and that the data collected with the measure meet the requirements of RMT (McKenna & Wilburn, 2018).

New HRQL measures have been developed that are specific to NF1 patients with pNFs (Draucker et al., 2017; Ferner et al., 2017; Lai et al., 2017; Nutakki et al., 2018). In contrast to those measures, the aim of this study was to develop and validate a measure of QoL specific to this patient group, employing the needs‐based model of QoL (Hunt & McKenna, 1992) and meeting the criteria for a high‐quality PRO.

## 2 METHODS

### 2.1 Ethical compliance

The UK study was granted ethics committee approval by the National Research Ethics Service Committee North West (14/NW/0279). In the US, approval was granted by the Johns Hopkins University School of Medicine (JHU‐SOM) Institutional Review Board.

### 2.2 Patients

In the UK, patients were recruited through the Children's Tumor Foundation Patient Registry (https://nfregistry.patientcrossroads.org) and the Genetics department at St Mary's Hospital, Manchester. For the postal validation survey, additional avenues of recruitment were utilised that included charities, support groups and additional clinical centres. Patients in the US were recruited from Johns Hopkins Comprehensive Neurofibromatosis Center, Baltimore and the Patient Registry. Patients at each stage provided written informed consent prior to inclusion in the study.

Eligibility requirements included; age of 18 or above, confirmed NF1 diagnosis, ≥1 pNF, ability to understand and complete questionnaires independently and ability to provide written informed consent. Patients were excluded if they were undergoing evaluation for malignant conversion of a pNF or had known active malignancy, were unable to provide informed consent or were deemed by the clinical team to be incapable of participating in the study. Patients with a history of malignancy that had been previously, curatively treated were included.

### 2.3 Stages in the development of the PlexiQoL

Three main stages were involved in the development of the PlexiQoL.

#### 2.3.1 Item generation

PlexiQoL content was derived from unstructured qualitative interviews conducted by experienced interviewers, with patients in the UK and US. Letters were sent to eligible patients inviting them to take part in the study. The UK interviews were conducted in a private room in either the Manchester Centre for Genomic Medicine at Saint Mary's Hospital, the offices of Galen Research or at the patient's home. US interviews were conducted at Johns Hopkins University, Baltimore. With the patient's permission, interviews were audio‐recorded and transcribed verbatim, with any patient‐identifying information removed from the transcripts to ensure anonymity.

The interviews focused on the ways in which pNFs and their treatment affected the ability of interviewees to fulfil their human needs. Interviewees were asked to describe how their condition impacted their everyday lives. Rather than asking specific questions, participants were encouraged to talk freely about whichever issues they considered important. Where participants reported symptoms or functional problems caused by their pNFs, they were asked to explain how these affected their ability to meet their needs.

Theoretical thematic analysis (Braun & Clarke, 2006), guided by the needs‐based model of QoL (Hunt & McKenna, 1992), was performed on the transcripts. Independent analysis of each transcript was conducted by two members of the research team (who had not interviewed the respondent), to identify potential QoL issues. All issues identified from the transcripts were recorded together and grouped by themes. The research team in the UK and a US NF clinician then worked together to refine the themes and to identify potential items, using the patients’ own words wherever possible. All themes were derived from the current transcripts only, with no reference made to themes generated in previous instrument development studies. An item pool was generated that contained items derived from both UK and US patients.

The draft questionnaire was produced containing the same items for both countries. However, the wording of some statements differed to reflect local language and idioms. The questionnaire was presented in a pen and paper format with “True”/”Not True” response options. Respondents were asked to base their responses on how they felt at the moment (UK) / at the present time (US). This format has proven to be the most effective in previous needs‐based measures and to be more sensitive than measures with multiple response options (De Jong et al., 1997).

#### 2.3.2 Assessment of face and content validity

Semi‐structured cognitive debriefing interviews (CDIs) were conducted with UK and US patients to determine the clarity, relevance, and applicability of the draft PlexiQoL. Patients were asked to complete the questionnaire in the presence of a researcher, who made detailed notes about hesitations or difficulties experienced by respondents. Guided by a semi‐structured interview schedule, the interviewer asked about the problems observed and specific questions about the suitability of the questionnaire content. Patients were encouraged to provide feedback on the items and instructions, and whether any aspects of their experience were not covered by the questionnaire. Interviewees were also asked how they referred to their pNFs. This was important to ensure that interviewees could distinguish these from dermal tumours and gliomas.

The research team analysed the CDI reports to identify and address any problematic items and/or instructions.

#### 2.3.3 Postal validation survey

A large scale postal survey was conducted in parallel in the UK and US to reduce the number of items in the PlexiQoL and to assess its scaling and psychometric properties.

Eligible participants were sent a questionnaire pack which included a demographic questionnaire, the draft PlexiQoL and a comparator measure. The comparator measure used in the UK was the Nottingham Health Profile (NHP; Hunt et al., 1981) and the Short Form‐36 (SF‐36; Ware & Sherbourne, 1992) was used in the US. These are generic measures of subjective health status (HRQL). The NHP comprises six sections; energy level, pain, physical mobility, sleep, social isolation, and emotional reactions. Each section is scored from 0 to 100, where 100 indicates the worst health state. The SF‐36 consists of 36 items, covering eight sections. Scores on the SF‐36 range from 0 to 100, with a higher score representing better health.

A subset of respondents was asked to complete the PlexiQoL approximately two weeks after the first administration to assess reproducibility.

##### Scale reduction

RMT is a measurement model that assesses whether a set of questions in a scale can be added together to provide a valid, unidimensional total score. It was used in this study to evaluate and improve the measurement properties of the draft PlexiQoL. A target sample size of 250 patients was selected. A sample of this size is required to provide over 99% confidence that the parameter estimates are stable within half a logit (Linacre, 1994). Where a scale fits the Rasch model it is unidimensional (measures one construct) and interval level measurement is achieved.

Internal reliability was assessed using the Person Separation Index (PSI). The PSI is indicative of the power of the items to distinguish between respondents. A PSI score of 0.70 is the minimum acceptable value (Tennant & Conaghan, 2007).

Fit of the PlexiQoL data to the model was investigated by reference to the overall item‐trait interaction Chi‐squarefit value. A significant Chi‐square statistic (*p* < 0.05) is indicative of misfit to the Rasch model. Item level fit was investigated via Chi‐square and Analysis of Variance (ANOVA) individual item fit statistics, in addition to individual item fit residuals. Statistical significance (*p* < 0.05) in the Chi‐square and F‐test indicates poor fit of an item to the model. Bonferroni adjustments were applied to these tests to account for multiple comparisons (Bland, 1995). Individual item fit residuals falling outside ±2.5 are indicative of model misfit.

A requirement of the Rasch model is that items should be invariant across groups. This is examined through tests of differential item functioning (DIF; Angoff, 1993). The groups examined for DIF were; age (below median versus above median), gender, and country. An ANOVA of standardized residuals was conducted, with a p‐value of <0.05 (Bonferroni corrections applied) considered indicative of the presence of DIF.

To be valid and reliable, items in a scale should be related but independent of each other. This is referred to as local independence and can be violated in two ways; multidimensionality and response dependency. The former is referred to as trait dependency, which occurs when a scale includes items that assess more than one construct. Response dependency occurs where the response to one item depends on the response to another (Marais & Andrich, 2008). Both types of local item dependency (LID) can be addressed by combining the dependent items into a single item, known as a subtest (Tennant & Conaghan, 2007). Item residual correlations of 0.2 above the average residual correlations for all items are considered indicative of a violation of local independence (Christensen et al., 2017).

Targeting of items to the respondents was assessed by examining person‐item distribution graphs. These show the ordering of both persons and items on the same logit scale and indicate whether the items in the scale are well matched to the respondents. Items with negative logit values are easier (more likely) to be affirmed by respondents. Items with positive logit values are more difficult (less likely) to be affirmed.

Rasch analysis was conducted using the RUMM2030 programme (Andrich et al., 2010).

##### Classical psychometric analysis

###### Internal consistency

Internal consistency measures the degree of relatedness of items. A Cronbach's alpha coefficient below 0.7 indicates that the items do not work together to form a scale (Streiner & Norman, 1995).

###### Test‐retest reliability

Test‐retest reliability is an estimate of a measure's reproducibility over time when no change in condition has taken place. This was assessed using Spearman's rank correlation to correlate PlexiQoL scores obtained on two different occasions. A value of 0.85 or above indicates that an instrument produces a low level of random measurement error (Weiner & Stewart, 1984).

###### Convergent validity

Convergent validity measures the level of association between scores on one scale and those on a comparator scale that measures a related construct. Scores obtained on the PlexiQoL were compared with NHP scores in the UK and SF‐36 scores in the US, using Spearman's rank correlation coefficients, to establish convergent validity.

###### Known group validity

Known group validity examines the ability of a measure to distinguish between groups of people that differ according to some known factor. Nonparametric tests for independent samples were employed to examine scores of respondents grouped by perceived pNF severity (mild, moderate, severe, very severe), perceived general health (very good, good, fair, poor), whether patients were taking pain medication and if they had pNFs that were visible to others.

PlexiQoL scores of respondents who differed by gender, age (above versus below median age), and country were also examined.

## 3 RESULTS

Demographic and disease information for participants at all stages of the study is shown in Table 1.

**TABLE 1:** Demographic information of UK and US patients involved at each stage of study.

	Qualitative interviews		Cognitive debriefing interviews			Validation survey	
	UK (n = 20)	US (n = 22)	UK (n = 16)	US (n = 15)		UK (n = 154)	US (n = 119)
Age (years)							
Mean (SD)	42.3 (13.4)	40.2 (11.4)	31.3 (9.0)		35.5 (11.5)	38.9 (13.2)	46.4 (14.0)
Range	20.7‐69.2	22.4‐63.9	18.1‐49.3		23.0‐64.3	18.2 ‐ 74.6	19.2 ‐ 79.9
Missing	1	1	1		0	0	2
Gender (%)							
Male	9 (45.0)	12 (54.5)	5 (31.3)		4 (26.7)	62 (40.3)	52 (43.6)
Female	11 (55.0)	10 (45.4)	11 (68.8)		10 (66.7)	92 (59.7)	66 (55.4)
Missing	0	0	0		1	0	1
Marital status (%)							
Married/living as	10 (50.0)	14 (63.6)	8 (50)		5 (33.3)	61 (39.6)	50 (42.0)
Divorced/separated	0	0	0		0	8 (5.2)	9 (7.6)
Widowed	0	0	0		0	1 (0.6)	3 (2.5)
Single	9 (45.0)	7 (31.8)	8 (50)		10 (66.7)	84 (54.5)	56 (47.0)
Missing	1	1	0		0	0	1
Work status (%)							
Full time employment	11 (55.0)	13 (59.1)	7 (43.8)		9 (60.0)	62 (40.3)	51 (42.9)
Part time employment	1 (5.0)	3 (13.6)	1 (6.3)		1 (6.7)	16 (10.4)	11 (9.2)
Retired	3 (15.0)	1 (4.5)	2 (12.5)		2 (13.3)	15 (9.7)	16 (13.4)
Student	0	0	1 (6.3)		1 (6.7)	13 (8.4)	6 (5.0)
Homemaker	0	1 (4.5)	1 (6.3)		1 (6.7)	4 (2.6)	1 (0.8)
Unemployed	0	0	0 (0)		1 (6.7)	19 (12.3)	21 (17.6)
Long‐term sick leave	5 (25.0)	1 (4.5)	4 (25.0)		0	19 (12.3)	3 (2.5)
Other	0	3 (13.6)	0		0	6 (3.9)	8 (6.7)
Missing	0	0	0		0	0	2
Perceived general health (%)							
Very good	7 (35.0)	6 (27.3)	0		0	19 (12.3)	22 (18.5)
Good	4 (20.0)	11 (50.0)	10 (62.5)		3 (62.5)	53 (34.4)	58 (48.7)
Fair	5 (25.0)	2 (9.1)	3 (18.8)		10 (18.8)	59 (38.3)	35 (32.8)
Poor	4 (20.0)	3 (13.6)	3 (18.8)		2 (18.8)	19 (12.3)	2 (1.7)
Missing	0	0	0		0	4	2
Perceived pNF severity (%)							
Mild	4 (20.0)	10 (45.5)	6 (37.5)		8 (53.3)	42 (27.3)	43 (36.1)
Moderate	2 (10.0)	7 (31.8)	6 (37.5)		5 (33.3)	61 (39.6)	44 (37.0)
Severe	10 (50.0)	2 (9.1)	3 (18.8)		1 (6.7)	35 (22.7)	22 (18.5)
Very severe	4 (20.0)	3 (13.6)	1 (6.3)		0 (0)	10 (6.5)	5 (4.2)
Missing	0	0	0		1	6	5
Taking prescription pain medication (%)							
Yes	13 (65.0)	9 (40.9)	6 (37.5)		7 (46.7)	42 (27.3)	40 (33.6)
No	7 (35.0)	13 (59.1)	10 (62.5)		8 (53.3)	110 (71.4)	78 (65.5)
Missing	0	0	0		0	2	1
Other health problems (%)							
Yes	9 (45.0)	12 (54.5)	11 (68.8)		10 (66.7)	103 (66.9)	74 (62.2)
No	11 (55.0)	10 (45.5)	5 (31.2)		5 (33.3)	49 (31.8)	39 (32.8)
Missing	0	0	0		0	2	6
Duration of illness (years)							
Mean (SD)	26.8 (15.0)	31.0 (10.2)	24.2 (8.8)		29.0 (11.0)	—	—
Range	1.0‐54.0	6.0‐58.0	3.4‐36.2		1.3‐45.2	—	—
Missing	1	1	1		1	—	—

### 3.1 Item generation

Forty‐two qualitative interviews were conducted that lasted between 10 and 90 minutes. The length of each interview was largely dependent on participants’ disease severity and their insight into the condition. As expected from previous research (Wilburn et al., 2017), this number of interviews ensured that saturation of themes was achieved.

From the transcripts, 1,080 statements were identified that described the impact of the condition on the interviewees’ lives. Of these, 696 statements related to the impact of pNFs on need fulfilment. These impacts covered issues relating to appearance, relationships, independence, role fulfilment, and pleasure. There was good concordance between the issues raised by UK and US patients, supporting the assumption that needs are universal.

Items for the questionnaire are in the form of statements made by interviewees. Questionnaire respondents are asked to state whether each statement applies to their current situation (True / Not True). Consequently, all statements generated from interviews are potential items. Items considered to be problematic were removed from the item pool. These included items that were; duplicated, idiosyncratic, complex, covered more than a single issue or ambiguous. Some items with similar wording or that were addressing the same issue were retained, to allow patients to select the most appropriate wording for items at later stages of the study.

A 42‐item draft questionnaire was identified that included items common to interviewees in both countries and covered all of the relevant themes.

### 3.2 Assessment of face and content validity

Overall, patients felt that the draft instructions and items were clear, relevant and applicable. The time taken to complete the PlexiQoL ranged from 2 to 7 (mean =4.4, SD =1.3) minutes. From the qualitative interviews, it had become apparent that several interviewees found it difficult to distinguish between pNFs and other neurofibromas. In a similar vein, when answering the items, some respondents were thinking of other aspects of NF1 rather than pNFs specifically. The cognitive debriefing interviews were used to determine how these problems could be overcome. Specifically, interviewees were asked how they normally referred to their pNFs as opposed to dermal tumours. In the UK these issues were addressed by adapting the questionnaire instructions to refer specifically to “plexiforms,” the term preferred, and understood best by UK patients. Most US interviewees referred to their pNFs as “nerve tumors” and this term was adopted for the US questionnaire instructions. An explanation of what pNFs are was also added to the front‐page instructions on the UK questionnaire. A similar instruction was not considered necessary by the US NF clinicians.

Items containing the word “it” were changed to “the plexiforms” / “tumors” respectively, to ensure that patients only considered the pNFs when answering the items. Slight changes were made to the wording of four items to improve clarity. No items were deleted from the draft measure at this stage. For two items, the word “hate” was changed to “dislike” in the US version. This is because “hate” was perceived as being too strong in the US and it is colloquially weaker in the UK.

### 3.3 Postal validation survey

The first administration of the questionnaire pack was returned by 273 (UK: n = 154; US: n = 119) patients. At Time 2, 143 responses were collected (UK: n = 84; US: n = 59). Data from both countries were combined to determine the final PlexiQoL and to assess its scaling properties, reliability, and validity.

#### 3.3.1 Scale reduction

Seven participants were removed from the analysis as they did not respond to the items in a logical manner, as would be expected to occur with all questionnaires. This can only be determined by the application of RMT. Twenty‐four participants who produced extreme scores (answering all items “true” or all items “not true”) were automatically excluded from the present analyses. While such respondents provide valid scores in clinical studies, they did not provide any information about item ordering. Twenty‐four of the 42 items were removed from the draft questionnaire using an iterative process. Ten items were removed for the reason of item misfit. Fourteen were removed due to LID, which was predominately the result of alternative wordings being included in the draft questionnaire. An example of an item pair displaying LID was “I find the plexiform(s) ugly” and “I find the plexiform(s) not very nice to look at”. In this instance, the latter item was removed from the questionnaire as it did not contribute additional information. Two pairs of items exhibiting LID and covering similar issues were made into subtests.

No DIF associated with age, gender or country was found.

Overall fit statistics for the final PlexiQoL are shown in Table 2. All items fit the Rasch model and internal reliability was good (PSI = 0.84).

**TABLE 2:** PlexiQoL Final Rasch Fit statistics.

	Item‐trait interaction (Chi^2^)	Person‐Separation Index (PSI)	Item‐person interaction			
			Items		Persons	
			Mean	SD	Mean	SD
PlexiQoL (18 items)	0.29	0.84	−0.45	1.04	−0.28	0.74
Target values	>0.002	>0.70	0.00	1.00	0.00	1.00

The easiest and most difficult items to affirm in the PlexiQoL are shown in Table 3. The logit positions of the items represent the location on the underlying interval level measurement scale. It can be seen from the table that the item “I feel I have no control over my illness” was the most commonly affirmed. The item “I can't take care of myself” was affirmed less than any of the others.

**TABLE 3:** Easiest and most difficult PlexiQoL items to affirm.

Easiest items to affirm		Most difficult items to affirm	
Item description	Location	Item description	Location
I feel I have no control over my illness	−2.81	I can't take care of myself	2.42
I find the plexiform(s) ugly/I am very self‐conscious when people are near me *(subtest)*	−2.34	I am reluctant to leave the house	2.19
I cover up the plexiform(s)	−1.39	I feel dependent on others	1.10

**FIGURE 1:**
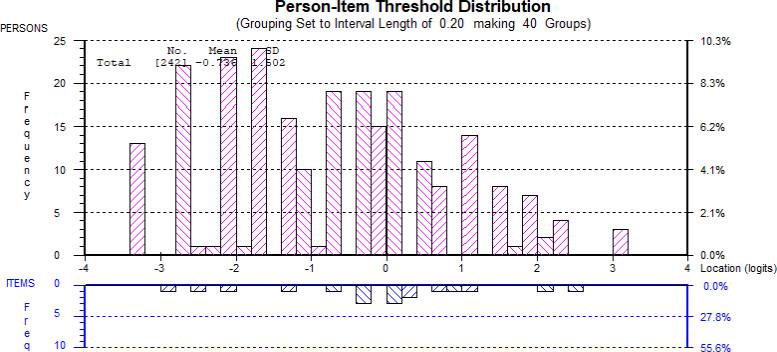
Location of PlexiQoL items and respondents on the Rasch interval scale.

Rasch analysis places respondents and items on the same measurement scale. The location of patients is shown in the top half of the figure and items in the bottom half. Figure 1 shows targeting of items to patients in the sample.

#### 3.3.2 Classical psychometric analyses

Descriptive statistics for scores on the 18‐item PlexiQoL scale, NHP and SF‐36 are shown in Table 4. A higher score on the PlexiQoL indicates worse QoL. As this was an instrument validation study, patients with one or more missing responses on the PlexiQoL were excluded from the psychometric analyses.

**TABLE 4:** Descriptive statistics for the postal validation survey.

	n	Median (IQR)	Range	% Scoring minimum	% Scoring maximum
PlexiQoL Time 1 (max n = 266)	234	7 (3‐10)	0‐18	7.3	2.1
NHP (max n = 149)					
Energy level	147	67 (0‐100)	0‐100	31.3	25.9
Pain	136	25 (0‐75)	0‐100	35.3	14.7
Emotional reactions	141	33 (0‐56)	0‐100	29.1	6.4
Sleep	143	40 (20‐80)	0‐100	23.8	13.3
Social isolation	145	20 (0‐60)	0‐100	47.6	9.0
Physical mobility	143	13 (0‐50)	0‐88	37.8	0.0
SF−36 (max n = 117)					
Physical functioning	112	80 (50‐95)	5‐100	0.0	23.2
Role limitations due to physical health	116	75 (25‐100)	0‐100	16.4	48.3
Role limitations due to emotional problems	117	100 (33‐100)	0‐100	16.2	59.0
Energy	112	45 (25‐65)	0‐100	0.9	0.9
Emotional well‐being	114	68 (40‐81)	4‐100	0.0	2.6
Social functioning	80	82 (50‐100)	0‐100	2.5	38.8
Bodily pain	112	58 (41‐82)	0‐100	6.3	14.3
General health	114	57 (35‐78)	0‐100	1.8	1.8
PlexiQoL Time 2 (max n = 139)	124	7 (3‐12)	0‐17	7.3	0.0

Abbreviations: IQR, inter quartile range; NHP, Nottingham health profile; SF‐36, 36‐Item Short Form Health Survey.

Some section scores on the SF‐36 appear high, suggesting that pNFs have a limited effect on health status. This is particularly the case with “role limitations due to emotional problems”. For this section, the median score was 100 with 59% of respondents indicating that they had no problems. Large end effects were observed for several NHP and SF‐36 sections. This indicates that the subscales are not well targeted to this sample. In contrast, minimal floor and ceiling effects were found for the PlexiQoL.

Cronbach's α coefficients for the PlexiQoL were 0.90 at both time points, confirming that the items had a good level of association. Test‐retest reliability was 0.90, demonstrating excellent reproducibility, indicating that the PlexiQoL produces low levels of measurement error.

Table 5 shows correlations between scores on the PlexiQoL and those on the comparator instruments. As the SF‐36 is scored in the opposite direction to the PlexiQoL and NHP, the correlations are negative. All sections in the comparator measures were moderately highly correlated with PlexiQoL scores indicating their influence on QoL.

**TABLE 5:** Correlation coefficients* between scores on the PlexiQoL, NHP sections, and SF‐36 domains.

	PlexiQoL
NHP	
Energy level	0.55
Pain	0.57
Sleep	0.52
Emotional reactions	0.72
Social isolation	0.69
Physical mobility	0.59
SF‐36	
Physical functioning	−0.52
Role limitations due to physical health	−0.51
Role limitations due to emotional problems	−0.57
Energy	−0.68
Emotional well‐being	−0.68
Social functioning	−0.63
Bodily pain	−0.49
General health	−0.65

Abbreviations: NHP, Nottingham Health Profile; SF‐36, 36‐Item Short Form Health Survey.

* All correlation significant at *p* < 0.01.

**FIGURE 2:**
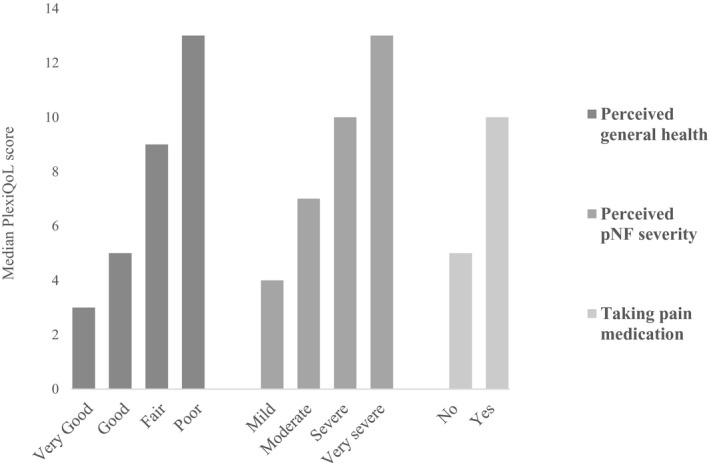
Median PlexiQoL scores by known groups.

Significant differences (*p* < 0.01) in PlexiQoL scores were observed for patients grouped by perceived general health, perceived pNF severity and use of pain medication (Figure 2).

These findings demonstrate that the PlexiQoL can distinguish successfully between groups of differing health status. The observed difference in PlexiQoL scores associated with the use of pain medication was explored further. A chi‐squared test of independence was performed to assess the relation between use of pain medication and perceived pNF severity. As expected, patients who rated their pNF severity as more severe, were more likely to be taking pain medication (χ^2^ (3, N = 254) = 27.1, *p* < 0.01).

It was expected that patients with visible pNFs would score higher on the PlexiQoL. However, a Mann–Whitney U test found no difference in PlexiQoL scores associated with the visibility of pNFs (*p* = 0.13).

Table 6 shows PlexiQoL scores for patients grouped by age (above or below median), gender, and country. No significant differences in PlexiQoL scores were found between participants grouped by these demographic variables.

**TABLE 6:** Associations between PlexiQoL scores and demographic factors.

	PlexiQoL	
	n	Median (IQR)
Age (*p* = .47)		
Below median	113	7 (3‐11)
Above median	119	6 (3‐10)
Gender (*p* = .12)		
Male	95	6 (2‐10)
Female	138	7 (3‐11)
Country (*p* = .25)		
UK	132	7 (3‐10)
US	102	5.5 (3‐10)

Abbreviation: IQR, inter quartile range.

## 4 DISCUSSION

The PlexiQoL is the first patient‐derived QoL measure specific to adults with NF1‐associated pNFs. It assesses the impact of pNFs on patients’ ability to fulfil their human needs. The scale adopts the needs ‐based model of QoL and joins a large portfolio of high‐quality outcome measures that are widely used in international clinical trials (see for example Marzo‐Ortega et al., 2005; McKenna et al., 2006; Tay et al., 2011). The scientifically rigorous methodology employed in the development of the PlexiQoL ensures accurate and valid measurement of the impact of the condition and the value of potential treatments to the lives of patients. It would be interesting for other researchers to confirm its performance in additional samples of NF1 patients with pNFs.

As the content of the PlexiQoL was generated directly from relevant adults with confirmed diagnoses, the measure is highly relevant to this patient group and captures their specific concerns. Results of the cognitive debriefing interviews indicated that the instrument was well accepted by patients, who felt that the items reflected their experience well. For example, one respondent stated that “I feel like I’m not alone” after completing the questionnaire. The measure consists of 18 dichotomous items and can be completed in fewer than five minutes. This allows for easy administration, completion and scoring.

The final version of the PlexiQoL fit the Rasch model and was free from DIF and local item dependency. Consequently, the measure provides a unidimensional index of outcome, rather than a profile of different types of outcome that cannot be validly combined. Fit to the Rasch model allows valid means and standard deviations to be calculated and the application of parametric statistical analyses (Tennant & Conaghan, 2007). Consequently, smaller sample sizes are required for clinical studies (Tennant et al., 2004). This advantage is accentuated by the high reproducibility demonstrated, meaning that low levels of measurement error are generated with the measure.

The PlexiQoL has a wide measurement range with 97.4% of respondents obtaining valid scores on the measure. Twenty‐four (8.8%) respondents scored at the extremes of the scale, which compares favourably with other PROMs. This again suggests that the scale will be able to detect true change resulting from effective interventions.

The moderately high correlations found between PlexiQoL scores and those on the NHP demonstrated that both the physical limitations and emotional impairments experienced by this patient group influence their QoL. Inconsistent scores were obtained with the SF‐36, in that respondents appeared to have better than expected health status.

The PlexiQoL was shown to be sensitive to differences in patient‐perceived pNF severity and general health and to the use of pain medication. The finding that there was no bias in PlexiQoL scores associated with age, gender, or country means that it is not necessary to control for these variables in clinical studies. The absence of DIF by country also confirms that respondents in the UK and US both considered pNFs, when answering the questionnaire.

It was expected that patients who had visible pNFs would have poorer scores on the PlexiQoL compared to those who could cover them. Assessment of pNF visibility was assessed by the question; “Are other people able to see that you have plexiform(s)?” Responses to this question appeared to be inconsistent as it was clear from responses that the question lacked the necessary specificity. A more objective indicator of visibility, possibly judged by a clinician, might prove more informative.

The PlexiQoL can be used in routine clinical practice and to evaluate the value gained by patients from interventions. As the measure is not treatment specific, the impact of both clinical and non‐clinical interventions can be determined using the measure. Future research is necessary to establish the responsiveness of the PlexiQoL – its ability to detect real changes in QoL resulting from interventions. This requires the availability of an established, effective intervention for the condition.

However, the responsiveness of a measure is dependent on it having a coherent measurement model, unidimensionality, construct validity and high reproducibility. A construct must be defined with precision, before it can be measured (Neale & Strang, 2015). Unidimensionality is a basic assumption of measurement theory and is essential for valid measurement (Segars, 1997). Finally, measures need excellent reproducibility to detect real change, as they will then have low levels of measurement error (Roach, 2006).

All these conditions are met by the PlexiQoL. Use of the measure in intervention studies will help determine the value patients gain from new and existing interventions for pNFs.

## CONFLICT OF INTEREST

This publication was supported by an Agreement from The Johns Hopkins University School of Medicine and the Neurofibromatosis Therapeutic Acceleration Program (NTAP). Its contents are solely the responsibility of the authors and do not necessarily represent the official views of The Johns Hopkins University School of Medicine.

## AUTHORS CONTRIBUTIONS

JW, SPM, JOB, and SH designed the study. JW, SPM, MR, and SL conducted the research. JW, AH, and MR performed the data analysis. AH and SPM wrote the manuscript. JOB, SL, and SH provided clinical input. All authors contributed to study conception, interpretation of data, and critical revision of the manuscript for important intellectual content.

## Data Availability

The data that support the findings of this study are available from the corresponding author upon reasonable request.
